# Association of Triglyceride-Glucose Index with Negative Clinical Outcomes in Geriatric Patients with Chronic Heart Failure

**DOI:** 10.3390/jcm15124794

**Published:** 2026-06-20

**Authors:** Li Tian, Xuan Qiu, Qiqi Cheng, Jun Shen, Suqing Wang

**Affiliations:** 1 School of Nursing, Wuhan University, Wuhan 430072, China; 2021283050150@whu.edu.cn; 2 Department of Cardiology, People’s Hospital, Hubei University of Medicine, Shiyan 442000, China; 13477301883@163.com; 3 Department of Cardiology, Xiangyang No. 1 People’s Hospital, Hubei University of Medicine, Xiangyang 441000, China; zhuchengcheng1118@163.com; 4 Department of Structural Heart Disease, Zhongnan Hospital of Wuhan University, Wuhan 430060, China; 2022283070023@whu.edu.cn

**Keywords:** TyG index, chronic heart failure, heart failure rehospitalization, all-cause mortality, Kaplan–Meier survival curve

## Abstract

**Objectives**: To determine the prognostic value of the triglyceride-glucose (TyG) index, which serves as a surrogate for insulin resistance, for heart failure rehospitalization and all-cause mortality in older adults with chronic heart failure, and to evaluate its clinical utility in risk stratification and nursing care. **Methods**: In this single-center retrospective cohort study, 786 patients aged ≥65 years with chronic heart failure hospitalized at a tertiary referral hospital in Central China (January 2022–January 2025) were included and divided into low vs. high TyG index groups based on the median. Baseline data were extracted from medical records. Follow-up ended in December 2025. Associations between TyG index and adverse outcomes were examined using Kaplan–Meier curves, restricted cubic spline (RCS) regression, and multivariable Cox proportional hazards models. **Results**: The median TyG index was 8.35. In unadjusted analyses, the high-TyG group had significantly greater cumulative risks of heart failure rehospitalization (*p* < 0.001) and all-cause mortality (*p* = 0.028). After multivariable adjustment, the TyG index remained independently associated with heart failure rehospitalization (hazard ratio [HR] = 1.63), whereas its association with all-cause mortality was attenuated and no longer significant. Restricted cubic spline analysis revealed a nonlinear dose–response relationship between the TyG index and heart failure rehospitalization, and a linear relationship with all-cause mortality. **Conclusions**: In elderly patients with chronic heart failure, the TyG index independently predicted heart failure rehospitalization and demonstrated a nonlinear dose–response relationship; its independent association with all-cause mortality was not significant after full adjustment. The index may nonetheless aid in risk stratification and individualized nursing in this population.

## 1. Introduction

Heart failure (HF) is a complex clinical syndrome caused by structural or functional abnormalities that impair ventricular filling or ejection, ultimately reducing pump efficiency. It represents the advanced stage of most cardiovascular diseases. The global prevalence and disease burden of HF continue to rise, a trend strongly driven by population aging that disproportionately affects older adults [1,2]. Systemic inflammation is a core pathophysiological feature of both acute and chronic HF [3]. Despite advances in pharmacotherapy and device-based interventions, elderly patients with HF remain at high risk of rehospitalization and mortality, imposing a heavy burden on healthcare systems [4,5,6,7,8]. In elderly patients with chronic heart failure, the prognosis remains poor despite advances in guideline-directed medical therapy. Epidemiological data indicate that the 30-day rehospitalization rate after discharge ranges from 14% to 22%, and the 1-year all-cause mortality rate is approximately 22–28% in this population [9,10]. These unfavorable outcomes highlight the urgent clinical need for simple, accessible, and reliable prognostic biomarkers to facilitate early risk stratification and personalized management. Recent studies further indicate that multiple HF rehospitalizations are strong independent predictors of mortality [11,12].

Insulin resistance (IR) is a key pathophysiological contributor to the development and progression of HF [13,14]. As recently highlighted by Trimarchi et al. [15], IR is closely intertwined with inflammatory activation in chronic HF. Metabolic triggers such as visceral adipose dysfunction drive this inflammatory process, thereby providing a mechanistic link between IR and its surrogate, the triglyceride-glucose (TyG) index, and adverse outcomes. This interplay promotes myocardial energy imbalance, oxidative stress, inflammation, and adverse cardiac remodeling, ultimately impairing cardiac function and worsening clinical outcomes [16]. However, direct measurement of IR is cumbersome, costly, and clinically impractical.

The triglyceride-glucose (TyG) index, calculated from fasting triglyceride and glucose concentrations, offers a convenient surrogate of insulin resistance using routinely available laboratory tests [17,18,19,20,21]. Accumulating evidence indicates that this index correlates with both the occurrence and prognosis of cardiovascular disorders [22,23,24,25,26]. In patients with chronic heart failure, several studies have reported that an elevated TyG index is associated with increased risks of adverse outcomes, including mortality and heart failure rehospitalization. However, evidence specifically focusing on elderly populations remains scarce, and findings regarding the shape of the dose–response relationship—whether linear or nonlinear—have been inconsistent. Moreover, data on long-term outcomes and the prognostic value of the TyG index specifically for heart failure rehospitalization in geriatric patients are particularly limited [24,25]. Nevertheless, data regarding its prognostic significance in older HF patients remain limited, particularly concerning nonlinear associations and long-term adverse events.

Therefore, this retrospective cohort study was conducted to evaluate the association between the TyG index and both HF rehospitalization and all-cause mortality in older adults aged ≥65 years with chronic HF (CHF). The aim was to identify a potential predictive biomarker for risk stratification and personalized treatment strategies. Determining the shape and strength of the TyG-adverse outcome association in this population may support the development of simple, cost-effective risk-stratification tools and inform personalized care strategies, including targeted nutritional and metabolic nursing interventions.

## 2. Subjects and Methods

### 2.1. Study Population

This retrospective cohort study enrolled eligible patients aged ≥65 years with a diagnosis of CHF admitted to a tertiary hospital in Hubei Province from 1 January 2022, to 31 January 2025.

Inclusion criteria: age ≥ 65 years; diagnosis of chronic heart failure CHF based on the 2021 European Society of Cardiology (ESC) Guidelines for the diagnosis and treatment of acute and chronic heart failure; complete baseline data and available follow-up information; sinus rhythm or atrial fibrillation with stable hemodynamics.

Exclusion criteria: acute myocardial infarction, severe liver or renal dysfunction, malignant tumors, or autoimmune diseases; missing key laboratory or follow-up data; loss to follow-up. For patients with multiple admissions, the first admission data were included.

### 2.2. Data Collection

Baseline demographic data and clinical characteristics BMI, comorbidities, and NYHA functional class, were collected; smoking and alcohol use history were also recorded. Venous blood samples were obtained after ≥8 h of overnight fasting. Standard biochemical parameters, including fasting blood glucose (FBG), triglycerides (TG), total cholesterol (TC), high-density lipoprotein cholesterol (HDL-C), low-density lipoprotein cholesterol (LDL-C), alanine aminotransferase (ALT), aspartate aminotransferase (AST), serum creatinine (Scr), uric acid (UA), N-terminal pro-B-type natriuretic peptide (NT-proBNP), cardiac troponin T (cTnT), were quantified using an automated biochemical analyzer Cobasc8000; Roche Diagnostics (Roche Diagnostics, Mannheim, Germany). Echocardiography Protocol: All enrolled patients underwent transthoracic echocardiography within 72 h after admission. Examinations were performed using a Philips EPIQ 7C ultrasound system (Philips Medical Systems, Andover, MA, USA) equipped with an S5-1 phased-array transducer (frequency 1.0–5.0 MHz). All procedures were independently conducted by two senior sonographers with more than 5 years of experience in cardiac ultrasound, strictly following the American Society of Echocardiography (ASE) guidelines. The measured parameters of cardiac structure and function included: left ventricular ejection fraction (LVEF, calculated using the Simpson biplane method), left atrial diameter (LA), left ventricular diameter (LV), interventricular septal thickness (IVST), left ventricular posterior wall thickness (LVPWT), right atrial diameter (RAD), and right ventricular diameter (RVD). All parameters were measured over three consecutive cardiac cycles, and the average value was used as the final result. All images were stored in the hospital picture archiving and communication system (PACS), and a random sample of measurements was reviewed by a third senior sonographer for quality assurance.

### 2.3. Calculation of the TyG Index

The TyG index was computed using the equation: TyG = ln(TG × FBG)/2, where TG and FBG were converted to mg/dL units. Participants were grouped into low and high TyG index strata based on the median TyG value.

### 2.4. Follow-Up and Outcomes

The primary endpoints were: heart failure rehospitalization and all-cause mortality. Participants were followed through outpatient records or telephone interviews until December 2025. The median follow-up duration was 22 months. Follow-up data were obtained through review of outpatient medical records and structured telephone interviews. All readmission and mortality outcomes were independently adjudicated by two senior cardiovascular physicians, who systematically reviewed the available medical charts, outpatient documentation, and follow-up information. In cases where the two reviewers did not reach an initial agreement, the outcome was referred to a third senior physician, whose decision was considered final. This independent adjudication procedure was employed to minimize ascertainment bias and to enhance the reliability of the study endpoints.

### 2.5. Statistical Analysis

Statistical analyses were performed using SPSS 25.0 and R 4.3.2. For continuous measures, parametric results were reported as mean ± standard deviation and compared with independent *t*-tests; non-parametric results were reported as median (interquartile range) and compared with the Mann–Whitney U test Categorical outcomes were described as frequencies (percentages) and assessed via chi-square tests. Time-to-event analyses were conducted using Kaplan–Meier survival curves and the log-rank test. Multivariable Cox regression was used to identify independent predictors. Nonlinear associations were examined with restricted cubic spline (RCS) analysis. All analyses adopted a two-sided significance criterion of *p* < 0.05.

### 2.6. Generative AI Tool

During the preparation and revision of this manuscript, we used ChatGPT (online version DS-V4-Pro) solely for English language editing, grammar, and logical refinement. The generative AI tool was not involved in study design, data collection, statistical analysis, interpretation of results, or drawing of conclusions. All content, viewpoints, and conclusions were independently prepared and reviewed by the authors. The authors have revised and verified all AI-assisted text and take full responsibility for the accuracy and academic integrity of the manuscript.

## 3. Results

### 3.1. Baseline Characteristics

The study cohort comprised 786 elderly patients with heart failure, evenly allocated to the low (*n* = 389) and high (*n* = 397) TyG index groups. Baseline demographic, clinical, laboratory and imaging indicators of participants in the two groups were systematically compared, and all detailed stratified data are summarized in Table 1. Marked intergroup differences were evident for age, sex, prevalence of diabetes, cTnT, FBG, TG, TC, HDL-C, LDL-C, AST, and the TyG index itself (all *p* < 0.05). In contrast, the two groups showed comparable profiles for body mass index (BMI), hypertension status, history of smoking or alcohol consumption, NT-proBNP, Scr, UA, ALT, and echocardiographic indices (all *p* > 0.05). The median TyG index for the entire cohort was 8.35.

### 3.2. Survival Analysis

**Figure 1:**
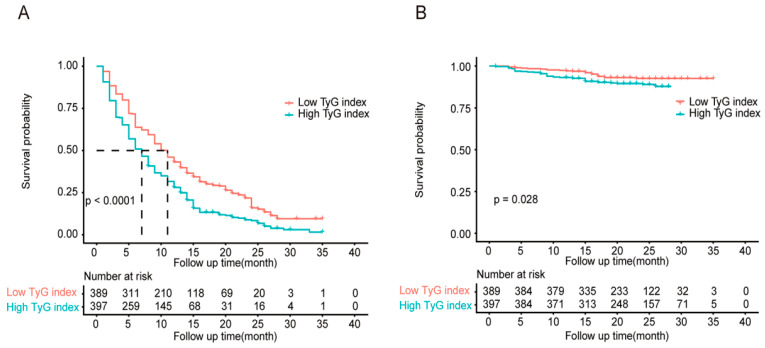
Kaplan–Meier curves depicting adverse clinical outcomes in heart failure individuals: (**A**) rehospitalization for heart failure; (**B**) all-cause mortality. The dashed black line in panel A marks the 10-month follow-up time point and 50% survival probability for comparing heart failure rehospitalization risk between the low-TyG and high-TyG groups.

Kaplan–Meier survival analysis revealed that, compared with the low-TyG index group, the high-TyG index group had a significantly higher cumulative incidence of both heart failure rehospitalization and all-cause mortality (Figure 1). The log-rank test confirmed a significant between-group difference for heart failure rehospitalization (log-rank *p* < 0.001) and for all-cause mortality (log-rank *p* = 0.028). In unadjusted Cox proportional hazards models, the high-TyG group was associated with a significantly increased risk of heart failure rehospitalization (HR = 1.57, 95% CI: 1.34–1.83) and all-cause mortality (HR = 1.72, 95% CI: 1.05–2.82) relative to the low-TyG group (Figure 1).

### 3.3. Cox Regression Analysis

To further support the findings from the Kaplan–Meier analysis, Cox proportional hazards regression models were used to assess the association between the TyG index and adverse outcomes. Four sequentially adjusted models were constructed: an unadjusted model; Model 1, adjusted for age and sex; Model 2, further adjusted for hypertension, diabetes mellitus, smoking, and alcohol use; and Model 3, additionally adjusted for NYHA functional class, NT-proBNP, and LVEF.

Multivariable Cox proportional hazards models demonstrated that a high TyG index, compared with a low TyG index, was independently associated with heart failure rehospitalization after sequential adjustment for confounders. This risk association proved consistent across all models: unadjusted (HR = 1.57, 95% CI: 1.34–1.83), Model 1 (HR = 1.59, 95% CI: 1.35–1.86), Model 2 (HR = 1.63, 95% CI: 1.39–1.92), and Model 3 (HR = 1.61, 95% CI: 1.36–1.90, *p* < 0.001).

For all-cause mortality, a high TyG index was associated with elevated risk in the unadjusted model (HR = 1.72, 95% CI: 1.05–2.82) and in Model 1 (HR = 1.65, 95% CI: 1.01–2.71). However, the association was attenuated and no longer statistically significant after full covariate adjustment in Model 2 (HR = 1.57, 95% CI: 0.95–2.59, *p* = 0.080) and remained non-significant in Model 3 (HR = 1.59, 95% CI: 0.96–2.69, *p* = 0.082) (Table 2).

### 3.4. Nonlinear Relationship Analysis

**Figure 2:**
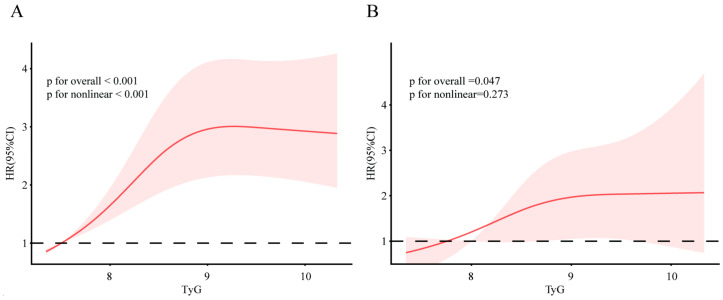
Restricted cubic spline curves of the prognostic correlation between TyG index and adverse cardiovascular events in elderly HF patients per ESC diagnostic criteria: (**A**) Heart failure rehospitalization; (**B**) All-cause mortality. The black dashed horizontal line represents the reference hazard ratio of 1; the solid red line shows the adjusted HR curve of TyG index; the red shaded area indicates the 95% confidence interval of HR estimates.

Restricted cubic spline (RCS) analysis was performed to evaluate the dose–response relationship between the TyG index and adverse outcomes, with adjustment for age, sex, hypertension, diabetes mellitus, smoking, and alcohol use (Figure 2). For heart failure rehospitalization, a significant nonlinear association was observed between the TyG index and rehospitalization risk (*p* for nonlinearity < 0.001). The hazard ratio for heart failure rehospitalization increased progressively with higher TyG values. For example, compared with a TyG index of 7.8 (10th percentile), a TyG index of 9.0 was associated with an HR of approximately 1.45 (95% CI:1.20–1.75), and a TyG index of 9.5 corresponded to an HR of approximately 1.70 (95% CI: 1.35–2.15). For all-cause mortality, the TyG index exhibited a linear relationship with mortality risk (*p* for nonlinearity = 0.295). Within the observed range, each 1-unit increase in the TyG index was associated with an HR of approximately 1.35 (95% CI: 1.02–1.79) for all-cause mortality, although the association was attenuated after full covariate adjustment as shown in Table 2.

### 3.5. Subgroup Analysis

Subgroup analyses stratified by demographics, lifestyle factors, and clinical characteristics consistently showed that a high TyG index was associated with an increased risk of heart failure rehospitalization across almost all examined strata, with no significant interactions observed (*p* for interaction >0.05). The elevated risk was evident both in patients aged 65–75 years (HR = 1.45, 95% CI: 1.17–1.79) and in those older than 75 years (HR = 1.93, 95% CI: 1.49–2.48), and it persisted irrespective of sex: men (HR = 1.84, 95% CI: 1.71–2.31) and women (HR = 1.51, 95% CI: 1.20–1.90, *p* < 0.001). Comparable patterns emerged when patients were grouped by hypertension and diabetes status. None of the interaction terms reached statistical significance, supporting the view that the prognostic implication of the TyG index for heart failure rehospitalization remains stable and uniform across these clinical subgroups (Table 3).

### 3.6. Formatting of Mathematical Components

TyG = ln(TG × FBG)/2(1)

where TG and FBG are expressed in mg/dL.

## 4. Discussion

### 4.1. Principal Findings

This study investigated the prognostic utility of the triglyceride-glucose (TyG) index—a readily obtainable surrogate marker of insulin resistance—in an elderly heart failure cohort. The principal findings are as follows: (1) a high TyG index was associated with substantially higher risks of heart failure rehospitalization and all-cause mortality compared with a low TyG index; (2) multivariable Cox regression confirmed that the association with heart failure rehospitalization was independent of conventional confounders; (3) restricted cubic spline analysis identified a nonlinear, dose-dependent pattern between the TyG index and rehospitalization risk; and (4) this association remained consistent across demographic and clinical subgroups, with no evidence of effect modification.

### 4.2. Comparison with Previous Literature

Prior investigations have established that the TyG index predicts incident cardiovascular events and mortality in the general population and in patients with coronary artery disease. However, data specifically addressing elderly heart failure populations remain sparse. Our findings extend this evidence by demonstrating that an elevated TyG index independently predicts rehospitalization in this aged heart failure cohort (HR = 1.63, 95% CI: 1.39–1.92), consistent with the meta-analysis by Khalaji et al. [25]. Compared with the study by Cheng et al. [24] in acute heart failure patients, the predictive effect in our study was more pronounced, likely because our study used long-term rehospitalization as the endpoint and included a high proportion (44.7%) of patients with diabetes. Furthermore, our subgroup analysis revealed that the prognostic value of the TyG index remained robust in patients with diabetes (HR = 1.69), suggesting its predictive effect may be independent of traditional metabolic risk factors. The robustness of the association between the TyG index and heart failure rehospitalization was further confirmed in an extended model (Model 3) that additionally adjusted for NYHA functional class, NT-proBNP, and LVEF (HR = 1.61, 95% CI: 1.36–1.90, *p* < 0.001). This finding indicates that the prognostic value of the TyG index for rehospitalization is independent not only of traditional cardiovascular risk factors but also of baseline heart failure severity and cardiac function. For all-cause mortality, the association remained non-significant after adjustment in Model 3 (HR = 1.59, 95% CI: 0.96–2.69, *p* = 0.082), further supporting the interpretation that the mortality risk associated with an elevated TyG index may be largely mediated by traditional risk factors and disease severity markers.

Using restricted cubic spline analysis, we revealed for the first time a nonlinear dose–response relationship between the TyG index and heart failure rehospitalization in an elderly chronic heart failure cohort (*p* for nonlinearity < 0.001). When the TyG index was relatively low (<8.5), rehospitalization risk increased slowly; when it exceeded approximately 8.5, the risk accelerated upward. This finding suggests that interpreting the TyG index as a continuous variable may be superior to using a fixed threshold for risk stratification.

The one-year all-cause mortality rate in our cohort (aged ≥ 65 years) was approximately 28%, consistent with the 28% reported by Kestens et al. [9] in hospitalized heart failure patients aged ≥75 years. Notably, the association between the TyG index and all-cause mortality attenuated to borderline significance after full covariate adjustment (HR = 1.57, 95% CI: 0.95–2.59, *p* = 0.080), suggesting that the mortality risk may be partially mediated by traditional risk factors such as age, diabetes, and hypertension.

### 4.3. Mechanistic Considerations

Although the underlying mechanisms cannot be directly inferred from this observational analysis, several pathways appear plausible. Insulin resistance, which the TyG index approximates, promotes myocardial metabolic inflexibility, endothelial dysfunction, oxidative stress, and systemic inflammation—processes that may accelerate heart failure progression. As recently highlighted by Trimarchi et al. [15], systemic inflammation is not merely a consequence but a core driver of chronic heart failure progression, serving as a critical biological link between metabolic abnormalities and myocardial injury.

Mechanistically, insulin resistance induces overexpression of pro-inflammatory cytokines (e.g., tumor necrosis factor-alpha and interleukin-6) and activates NF-κB signaling pathways, thereby exacerbating myocardial inflammation and oxidative injury. In the context of insulin resistance, increased release of free fatty acids from adipose tissue further activates Toll-like receptor 4-mediated inflammatory cascades, creating a vicious cycle of insulin resistance, inflammation, and myocardial injury. Therefore, an elevated TyG index may increase the risk of heart failure rehospitalization and mortality through these inflammation-related pathways. However, due to the limitations of our retrospective design, we were unable to include inflammatory biomarkers to directly verify this mediating effect.

### 4.4. Subgroup Consistency

Across predefined subgroups defined by age, sex, and comorbidity burden, the prognostic association of the TyG index remained stable, with no evidence of effect modification. This uniformity reinforces the external validity of our findings and supports the TyG index as a consistent risk stratification tool for elderly patients with heart failure.

### 4.5. Limitations

Several methodological limitations warrant consideration.

First, the retrospective, single-center design inherently precludes causal inference; the observed associations should be interpreted as hypothesis-generating. All participants were recruited from a single tertiary hospital in Hubei Province, and the cohort was predominantly of Han Chinese ethnicity, limiting generalizability to other regions or ethnic groups.

Second, despite rigorous adjustment for major confounders (age, sex, hypertension, diabetes, smoking, alcohol use, left ventricular ejection fraction, NYHA functional class, and NT-proBNP), residual confounding cannot be excluded. Notably, we were unable to adjust for key medication variables (loop diuretics, RAAS inhibitors, beta-blockers, SGLT2 inhibitors) due to the retrospective design, as post-discharge medication adjustments were not systematically documented and medication use is time-dependent.

Third, the TyG index was measured only at baseline, precluding assessment of its dynamic changes and their impact on prognosis.

Fourth, outcome data were primarily derived from outpatient medical records and telephone follow-ups. Although we implemented an independent adjudication process (two senior cardiologists, with disagreements referred to a third), the sources of outcome information still carry potential bias, including under-ascertainment of rehospitalization events and recall bias for out-of-hospital deaths.

Fifth, the mortality endpoint was defined as all-cause death, and we could not distinguish between cardiac and non-cardiac death.

Sixth, this study did not include inflammatory biomarkers (e.g., hs-CRP, IL-6), limiting our ability to explore the underlying biological mechanisms.

Seventh, the relatively limited number of all-cause mortality events may have resulted in insufficient statistical power in the fully adjusted model, potentially masking a modest but true association.

### 4.6. Future Directions

Despite these limitations, our findings provide important directions for future research. Larger-sample, multicenter, prospective cohort studies are needed to validate our findings. We aim to establish an optimal risk stratification cutoff for the TyG index using ROC curve analysis with the Youden index in independent external validation cohorts. Future studies should incorporate standardized documentation of medication regimens, inclusion of inflammatory biomarkers, and independent clinical endpoint adjudication to provide higher-quality evidence.

### 4.7. Clinical Implications and Conclusions

From a clinical perspective, the TyG index is easily derived from routine laboratory parameters, enhancing its feasibility for broad application in older patients with heart failure. This study reveals that an elevated TyG index independently confers a greater risk of heart failure rehospitalization among elderly patients, with a nonlinear dose–response relationship. While the link with all-cause mortality was attenuated after full covariate adjustment, a discernible gradient toward higher mortality risk persisted. Given its simplicity, affordability, and broad accessibility, the TyG index holds promise as a practical biomarker for identifying elderly heart failure patients at heightened risk of adverse outcomes. Future large-scale prospective studies are warranted to corroborate these observations and clarify the underlying mechanisms.

## 5. Conclusions

This study reveals that an elevated TyG index independently confers a greater risk of heart failure rehospitalization among elderly patients with HF. The prognostic association for recurrent events was consistent across major clinical subgroups and exhibited a nonlinear dose–response relationship, reinforcing its potential as a dependable marker for risk stratification. While the link with all-cause mortality was attenuated following full covariate adjustment, a discernible gradient toward higher mortality risk in patients with raised TyG levels persisted. Given the simplicity, affordability, and broad accessibility of its component measurements, the TyG index holds promise as a practical biomarker for identifying elderly HF patients at heightened risk of adverse outcomes. Future large-scale prospective studies are warranted to corroborate these observations and clarify the mechanisms by which insulin resistance, as reflected by the TyG index, contributes to disease progression in this vulnerable population.

## Figures and Tables

**Table 1:** Baseline Cohort Characteristics Stratified by Triglyceride-Glucose (TyG) Index Groups.

Variable	Low-TyG Group (*n* = 389)	High-TyG Group (*n* = 397)	F/χ^2^/Z	*p*-Value
Age, years	74.26 ± 9.38	72.84 ± 9.65	2.103	0.036
Male, *n* (%)	197 (50.64)	222 (55.92)	4.378	0.036
BMI, kg/m^2^	23.31 (20.96–25.15)	23.00 (21.24–24.48)	−1.074	0.238
Hypertension, *n* (%)	153 (39.33)	156 (39.29)	0.001	0.986
Diabetes mellitus, *n* (%)	134 (34.45)	217 (54.66)	32.480	<0.001
Smoking history, *n* (%)	54 (13.88)	70 (17.63)	2.036	0.154
Drinking history, *n* (%)	46 (11.83)	65 (16.37)	3.350	0.067
NYHA class			2.718	0.257
Class I–II	74 (19.02)	61 (15.37)		
Class III	183 (47.04)	183 (46.10)		
Class IV	132 (33.93)	153 (38.54)		
NT-proBNP, pg/mL	4216.00 (1782.00–9501.00)	3923.00 (1816.50–8752.50)	−1.032	0.302
cTnT, ng/L	0.024 (0.016–0.041)	0.031 (0.016–0.061)	−3.530	<0.001
Scr, μmol/L	93.90 (74.80–114.00)	92.80 (75.20–122.30)	−0.903	0.367
UA, μmol/L	381.70 (317.60–467.70)	383.50 (316.20–491.25)	−0.184	0.854
FBG, mmol/L	5.66 (4.96–6.47)	8.34 (6.57–10.58)	−15.425	<0.001
TG, mmol/L	0.71 (0.55–0.88)	1.38 (1.05–1.84)	−19.514	<0.001
TC, mmol/L	2.84 (2.30–3.59)	3.64 (2.85–4.34)	−8.363	<0.001
HDL-C, mmol/L	0.99 (0.81–1.23)	0.90 (0.76–1.15)	−2.768	0.006
LDL-C, mmol/L	1.57 (1.16–2.11)	2.26 (1.49–2.90)	−9.737	<0.001
AST, U/L	20.00 (17.00–27.60)	20.00 (18.00–32.25)	−2.616	0.009
ALT, U/L	17.00 (11.00–25.90)	18.00 (10.80–29.40)	−1.063	0.288
LVEF, %	46.50 (38.00–57.00)	47.00 (38.00–56.00)	−0.686	0.493
LA, mm	45.00 (41.00–51.00)	45.00 (41.00–50.00)	−0.942	0.346
LV, mm	51.00 (50.00–55.00)	51.00 (50.00–55.00)	−0.656	0.512
IVST, mm	11.00 (10.00–11.00)	11.00 (10.00–11.50)	−0.836	0.403
LVPWT, mm	10.00 (10.00–11.00)	10.00 (10.00–11.00)	−0.484	0.628
RAD, mm	26.00 (20.00–30.00)	26.00 (20.00–30.00)	−0.606	0.545
RVD, mm	20.00 (16.00–23.00)	20.00 (16.00–23.00)	−0.616	0.538
TyG index	8.09 (7.86–8.34)	9.04 (8.80–9.43)	−24.263	<0.001

Table Note 1: Abbreviations: ALT, alanine aminotransferase; AST, aspartate aminotransferase; BMI, body mass index; cTnT, cardiac troponin T; FBG, fasting blood glucose; HDL-C, high-density lipoprotein cholesterol; IVST, interventricular septal thickness; LDL-C, low-density lipoprotein cholesterol; LV, left ventricular diameter; LVEF, left ventricular ejection fraction; LVPWT, left ventricular posterior wall thickness; NT-proBNP, N-terminal pro-B-type natriuretic peptide; NYHA, New York Heart Association functional classification; RAD, right atrial diameter; RVD, right ventricular diameter; Scr, serum creatinine; TC, total cholesterol; TG, triglycerides; TyG, triglyceride-glucose index; UA, uric acid. The F/χ^2^/Z column presents the test statistics as follows: F for independent *t*-test (normally distributed continuous variables); Z for Mann–Whitney U test (non-normally distributed continuous variables); χ^2^ for χ^2^ test (categorical variables). Normally distributed continuous variables are presented as mean ± SD; non-normally distributed continuous variables are presented as median (IQR); categorical variables are presented as *n* (%).

**Table 2:** Prognostic Significance of the TyG Index for Cardiovascular Outcomes in Patients with HF.

	Low-TyG	High-TyG	*p*-Value
Heart failure rehospitalization			
Unadjusted	Ref	1.57 (1.34–1.83)	<0.001
Model 1	Ref	1.59 (1.35–1.86)	<0.001
Model 2	Ref	1.63 (1.39–1.92)	<0.001
Model 3	Ref	1.61 (1.36–1.90)	<0.001
All-cause mortality			
Unadjusted	Ref	1.72 (1.05–2.82)	0.030
Model 1	Ref	1.65 (1.01–2.71)	0.046
Model 2	Ref	1.57 (0.95–2.59)	0.080
Model 3	Ref	1.59 (0.96–2.69)	0.082

Table Note 2. Unadjusted model: no covariates. Model 1: adjusted for age and sex. Model 2: additionally adjusted for hypertension, diabetes mellitus, smoking, and alcohol use. Model 3: additionally adjusted for NYHA functional class, NT-proBNP, and LVEF. Ref: Reference group. Data are presented as hazard ratio (95% confidence interval).

**Table 3:** Stratified Analysis of the TyG Index as a Predictor of Heart Failure Rehospitalization and All-Cause Mortality.

	HR (95% CI)	*p*-Value	*p* for Interaction
Heart failure rehospitalization			
Age, years			0.184
65–75	1.45 (1.17–1.79)	0.001	
>75	1.93 (1.49–2.48)	<0.001	
Sex			0.067
Male	1.84 (1.71–2.31)	<0.001	
Female	1.51 (1.20–1.90)	<0.001	
Hypertension			0.351
Yes	2.08 (1.59–2.70)	<0.001	
No	1.46 (1.19–1.79)	<0.001	
Diabetes mellitus			0.195
Yes	1.69 (1.32–2.16)	<0.001	
No	1.66 (1.34–2.06)	<0.001	
Smoking			0.813
Yes	2.26 (1.43–3.56)	<0.001	
No	1.62 (1.36–1.93)	<0.001	
Drinking			0. 127
Yes	2.86 (1.75–4.66)	<0.001	
No	1.59 (1.34–1.89)	<0.001	
All-cause mortality			
Age, years			0.354
65–75	1.73 (0.86–3.48)	0.124	
>75	1.56 (0.74–3.28)	0.246	
Sex			0.715
Male	2.19 (0.97–4.96)	0.061	
Female	1.48 (0.78–2.81)	0.234	
Hypertension			0.029
Yes	1.54 (0.82–2.90)	0.553	
No	1.97 (1.00–3.91)	0.051	
Diabetes mellitus			0.642
Yes	2.02 (0.99–4.14)	0.436	
No	1.36 (0.68–2.72)	0.390	
Smoking			0.471
Yes	1.82 (1.07–3.10)	0.022	
No	0.30 (0.05–1.78)	0.186	
Drinking			0.294
Yes	0.56 (0.13–2.51)	0.449	
No	1.84 (1.07–3.16)	0.027	

Table Note 3. HR, hazard ratio; CI, confidence interval. *p* for interaction was assessed by a likelihood ratio test comparing models with and without the interaction term. All statistical tests were two-sided, with *p* < 0.05 considered statistically significant.

## Data Availability

The data that support the findings of this study are not publicly available due to privacy and ethical restrictions, as they contain potentially identifying patient information. However, the data can be made available from the corresponding author upon reasonable request, subject to the approval of the Ethics Committee of Zhongnan Hospital of Wuhan University.

## Abbreviations

The following abbreviations are used in this manuscript:

ACM	All-Cause Mortality
ALT	Alanine Aminotransferase
AST	Aspartate Aminotransferase
BMI	Body Mass Index
CHF	Chronic Heart Failure
CI	Confidence Interval
cTnT	Cardiac Troponin T
ESC	European Society of Cardiology
FBG	Fasting Blood Glucose
HDL-C	High-Density Lipoprotein Cholesterol
HF	Heart Failure
HR	Hazard Ratio
IR	Insulin Resistance
IVST	Interventricular Septal Thickness
LA	Left Atrial Diameter
LDL-C	Low-Density Lipoprotein Cholesterol
LV	Left Ventricular Diameter
LVEF	Left Ventricular Ejection Fraction
LVPWT	Left Ventricular Posterior Wall Thickness
NT-proBNP	N-terminal Pro-B-type Natriuretic Peptide
NYHA	New York Heart Association Functional Classification
RAD	Right Atrial Diameter
RCS	Restricted Cubic Spline
RVD	Right Ventricular Diameter
Scr	Serum Creatinine
TC	Total Cholesterol
TG	Triglycerides
TyG	Triglyceride-Glucose Index
UA	Uric Acid
